# Editorial: Bioinformatics Tools (and Web Server) for Cancer Biomarker Development

**DOI:** 10.3389/fonc.2020.599085

**Published:** 2020-10-20

**Authors:** Longxiang Xie, Liuyang Wang, Wan Zhu, Jing Zhao, Xiangqian Guo

**Affiliations:** ^1^Cell Signal Transduction Laboratory, Department of Preventive Medicine, Bioinformatics Center, Henan Provincial Engineering Center for Tumor Molecular Medicine, Institute of Biomedical Informatics, School of Basic Medical Sciences, Henan University, Kaifeng, China; ^2^Department of Molecular Genetics and Microbiology, School of Medicine, Duke University, Durham, NC, United States; ^3^Department of Anesthesia, Stanford University, Stanford, CA, United States; ^4^Department of Pathophysiology, Chongqing Medical University, Chongqing, China

**Keywords:** bioinformatics, webserver, prognostic, biomarker, TCGA, GEO, RNA sequence

Cancer remains a severe public health burden globally. The identification of molecular biomarkers play significant roles in diagnosis, treatment and prognosis of human cancers (1). Up to now, the tumor molecular heterogeneity and lack of sufficient biomarkers are two of the major difficulties in cancer treatment and prognostication. With the advance of recent development of high-throughput microarray and sequencing technologies, the public cancer transcriptomic databases, including The Cancer Genome Atlas (TCGA) and Gene Expression Omnibus (GEO), have increased dramatically (2). These databases offer additional resources and opportunities for biomarker discovery and validation (2). Unfortunately, those resources are not efficiently explored, and translation of stored high dimension data into clinical use are not feasible for clinicians and basic researchers without much bioinformatics background. Therefore, the user-friendly online web servers/tools are urgently needed for researchers. In this Research Topic, we have collected a series of original research articles and reviews, providing a number of useful web resources and tools. Those tools will facilitate better and accurate discovery of cancer biomarkers and expedite their clinical translation.

Currently, several powerful bioinformatics webservers/tools, such as KM plotter, GEPIA (Gene Expression Profiling Interactive Analysis), Oncomine and TIMER (Tumor Immune Estimation Resource), have been developed to analyze the public transcriptomic datasets along with clinical information for oncology research (3–6). However, limitations are still present for these webservers/tools, such as tedious registration process or single data source. To overcome these limitations, Yan et al. developed a new survival analysis web-server OSluca for lung cancer based on 5,245 clinical samples from TCGA, GEO and Roepman study. With OSluca, the users are able to assess the prognostic value of gene of interest, and the results will be presented by Kaplan-Meier (KM) plot, Hazard ratio (HR), and log-rank *p*-value. Dong et al. also collected 684 samples with long-term follow-up clinical information from 7 TCGA, GEO and Chinese Glioma Genome Atlas (CGGA) datasets, and developed a survival analysis online tool OSgbm for glioblastoma. In recent years, T cell repertoire sequencing (TCRSeq) data have been rapidly developed, however, tools for comprehensive analysis and visualization of TCR-Seq data have not been developed. Ni et al. developed a tool called VisTCR (Visual TCRSeq), an interactive software with a graphical user interface (GUI) for TCR data management, short-read sequence mapping, and post-analysis of TCR clonotype. VisTCR can be used to perform clonotype extraction and downstream analyses within a single data management framework, which will greatly help TCRseq data management and analysis in cancer immunotherapy. In a review of webserver/tools for cancer prognosis analysis, Zheng et al. described 22 webservers/tools for survival analysis based on mRNA, ncRNA, DNA and protein data, including LOGpc, KM plotter, GEPIA, OncoLnc, TCPA, MethSurv, PrognoScan, SurvExpress, and UALCAN, and they also gave a detailed description of the software usage, characteristics and algorithms of all these tools. They also discussed several major challenges and future directions in this area.

Those online webservers/tools for survival analysis would help clinician and researchers to discover novel prognostic biomarkers (3–6), to find the important therapeutic targets, and to investigate the potential molecular mechanisms of tumorigenesis and progression. Using a series of online databases, such as Oncomine and GEPIA, Kaplan-Meier plotter, TCGA, and cBioPortal, Sun et al. systematically analyzed the expression variation and prognostic value of sirtuins (SIRTs) 1–7 in ovarian cancer. The bioinformatics analysis showed that SIRT1-4, 6 and 7 may be novel prognostic biomarkers. Zhu et al. used a range of online tools, including Oncomine, GEPIA, TISIDB, and Kaplan-Meier plotter, to evaluate the expression and prognostic value of *CD38*. The results showed that compared with normal ovarian tissue, *CD38* is highly expressed in epithelial ovarian cancer (EOC), and higher *CD38* expression is associated with better prognosis. In addition, *CD38* was found to be associated with tumor-infiltrating lymphocytes (TILs), especially with activated CD8C T cells by TIMER. This implies the vital immunoregulatory role of *CD38* in the EOC microenvironment, and provides a novel prognostic biomarker and potential immunotherapy target. Yu et al. assembled 45,313 pancreatic cancer-specific AS (Alternative splicing) events of 10,623 genes from the TCGA and SpliceSeq database, and performed the cox univariate analyses of overall survival (OS). They found 6,711 AS events are remarkably associated with OS in pancreatic cancer. Notably, AS events of five genes including *DAZAP1, RBM4, ESRP1, QKI*, and *SF1*, were found to be significantly correlated with OS. Using the DriverDBv2, 13 driver genes were identified correlated with survival-associated AS events, including *TP53* and *CDC27*. These findings uncover that the aberrant AS patterns might serve as prognostic predictors in pancreatic cancer. Ding et al. performed the comprehensive characterization of differentially expressed genes between 65 normal colon tissues and 74 CRC samples, and identified 20 hub genes with a high degree of connectivity from the protein–protein interaction (PPI) network. Furthermore, knockdown of one hub gene, *MAD2L1*, significantly inhibited the CRC cell growth by impairing cell cycle progression and inducing cell apoptosis, implying that *MAD2L1* could be as a novel potential biomarker for diagnosis and therapy in CRC.

Single nucleotide polymorphism array (SNP-A) detects population-level genomic polymorphisms and chromosomal abnormalities such as submicroscopic or cryptic deletions or duplications (7). Xiao et al. used SNP-A technique to investigate the chromosomal abnormalities in 350 myelodysplastic syndromes (MDSs) patients and 26 healthy individuals. They showed that chromosomal aberrations contributed to a unfavorable prognosis in patients with myelodysplastic syndromes, and were closely related with an increased risk of transformation to typical myelodysplastic syndrome in patients with idiopathic cytopenia of undetermined significance. Thus, SNP-A can help assess the prognosis of patients with MDSs and the risk of disease progression for patients with ICUS.

Engineered organoids with sequential introducing driver mutations can provide important new clues for studying the mechanisms of cancer progression. Ping et al. developed an comprehensive strategy to capture the dynamic progression of CRC and prioritize gene cascading paths to model CRC through engineered organoids. From the single-mutant to quintuple-mutant engineered organoids, they characterized the functional activities of hallmark signatures and filled the substantial biological gaps between the engineered organoids and the CRC samples.

Although many single-gene cancer biomarkers have been reported, multi-gene signatures capture more information and may be more powerful for cancer prognosis, and they can be developed by analyzing public microarray data and RNA sequencing data (8). Based on the TCGA database and weighted gene co-expression network analysis (WGCNA), Tang et al. used Kaplan-Meier survival analysis and multivariate Cox regression method, and identified a four-gene prognostic signature (*CLEC5A, FMOD, FKBP9, LGALS8*) that was related with OS and recurrence time of 524 GBM patients. Those signature genes divided GBM patients into high-risk and low-risk groups, and the 5-years survival rate of the low-risk group was significantly higher than that of the high-risk group. Yang et al. profiled 4 GEO datasets and TCGA dataset from GBM patients, and performed the differential expression analysis, WGCNA and Cox regression analysis to identify core genes associated with clinical outcomes. A four-gene prognostic signature (*SLC12A5, CCL2, IGFBP2*, and *PDPN*) that was able to divide GBM patients into high-risk and low-risk groups. High-risk group showed higher mortality than low risk group by Kaplan–Meier curve. Yang et al. obtained 502 differential expressed miRNAs based on miRNA expression profiles of CRC patients from TCGA. Among these miRNAs, a novel five-miRNA signature (hsa-miR-5091, hsamiR-10b-3p, hsa-miR-9-5p, hsa-miR-187-3p, hsa-miR-32-5p) that could predict OS of CRC patients was constructed, verified and assessed in training group, testing group, and entire cohort. Furthermore, univariate and multivariate cox regression analysis showed that the five-miRNA signature could serve as an independent prognostic factor in CRC. Wang et al. investigated the expression profile of 63 central carbon metabolism–associated genes in 514 diffuse low-grade glioma cases (astrocytoma, oligodendroglioma, and oligoastrocytoma) from TCGA, and explored the prognostic roles of individual genes and the multiple-gene combination by Kaplan–Meier curve and multivariate cox regression analysis. The results showed that a four genes-signature (*RAF1, AKT3, IDH1*, and *FGFR1*) is positively associated with OS in patients with astrocytoma, suggesting that multigene expression signature is able to predict the prognosis of low-grade glioma patients.

Increasing studies have demonstrated that the competitive endogenous RNAs (ceRNA) regulation network plays an important role in cancer development (9). Yu et al. used WGCNA to construct the lncRNA co-expression networks, miRNA co-expression networks, and mRNA co-expression networks based on TCGA-ESCC RNAseq data. They identified 21 hub lncRNAs, seven hub miRNAs, and nine hub mRNAs, and constructed a ceRNA network, the similar ceRNA network was also built for head and neck squamous cell carcinoma (HNSCC) by using UALCAN, OncomiR and OncoLnc webtools. Two hub genes including *TBC1D2* and *ATP6V0E1* were found to be associated with the survival time of HNSCC. The ceRNAs network might provide common mechanisms involving in ESCC and HNSCC. The same group also constructed the gene co-expression networks and miRNA co-expression networks in Idiopathic pulmonary fibrosis (IPF) based on two GEO datasets (GSE3257 and GSE3258), then validated the clinical significance of the genes and the miRNAs in other three GEO datasets (GSE10667, GSE70866, and GSE27430). They identified seven hub miRNAs and six hub mRNAs, and constructed an interaction network of hub miRNAs-hub genes, which was also analyzed in non-small cell lung cancer (NSCLC). In addition, six hub genes and three miRNAs were found to be associated with the survival time of lung adenocarcinoma (LUAD).

The increasing multi-omics data greatly help us to understand cancer biology and identification of molecular biomarkers, but add additional layers of difficulty in data processing and analyses. In this special issue, a range of powerful bioinformatics tools/webservers for data analysis have been developed, and they will easily assist clinical and basic science researchers in biomarker development and validation. Of note, the bioinformatics tools/web servers presented here still need lots of improvements, for example, integrating the tumor tissue image, multi-omics network mapping, multi-gene signature assessment, and nomogram construction. After tackling these problems in future, the bioinformatics tools/webservers will be more powerfully for discovering cancer biomarkers and innovative cancer therapies.

## Author Contributions

All authors listed have made a substantial, direct and intellectual contribution to the work, and approved it for publication.

## Conflict of Interest

The authors declare that the research was conducted in the absence of any commercial or financial relationships that could be construed as a potential conflict of interest.
